# Nitrous Oxide

**Published:** 1867-12

**Authors:** 


### NITROUS OXIDE.

### LIVERPOOL ROYAL INSTITUTION.

On Thursday evening, Nov. 21st, the members of the Liverpool Chemists' Association assembled to hear a Lecture from Dr. Waite, Dentist, on "Anæsthesia: Nitrous oxide as a substitute for chloroform and ether," illustrated by experiments. Dr. Waite described several modes of inducing both local and general insensibility to pain, including Dr. Richardson's ether spray, etc., and stated that we are indebted to the Dental profession for the first discovery of a process of inducing anæsthesia. In September, 1844, Dr. Horace Wells, a Dentist of Hartford, Connecticut, was the first individual to undergo an operation, and also to perform operations in the anæsthetic state, and the agent he employed was nitrous oxide gas. This gas when mixed with air was of a very exciting and intoxicating nature, but when breathed undiluted with air it rapidly induced a peaceful and highly agreeable state of repose, accompanied by insensibility to pain. It closely resembled air in its chemical characteristics, and was therefore the most innocent and least injurious agent ever employed. Its administration was not accompanied by any unpleasant results, such as too often attended the use of chloroform and ether, and it had been employed almost exclusively throughout the United States for some two years, in preference to all other agents, and hitherto with none of the fatal consequences to which chloroform was particularly liable. The lecturer claimed for his profession the honor of having conferred a priceless boon upon suffering humanity, by the discovery of anæsthesia, and trusted that we should soon be able to discard the injurious agents now commonly employed, and so free the humane process of operating under anæsthesia from the objections which now attend it. The lecture was well received, and at the close some interesting experiments as to the anæsthetic properties of nitrous oxide were made.—A vote of thanks was passed to Dr. Waite for his instructive paper.

